# Machine learning algorithms for diagnosis of hip bone osteoporosis: a systematic review and meta-analysis study

**DOI:** 10.1186/s12938-023-01132-9

**Published:** 2023-07-10

**Authors:** Fakher Rahim, Amin Zaki Zadeh, Pouya Javanmardi, Temitope Emmanuel Komolafe, Mohammad Khalafi, Ali Arjomandi, Haniye Alsadat Ghofrani, Kiarash Shirbandi

**Affiliations:** 1 Department of Anesthesia, Cihan University - Sulaimaniya, Sulaymaniyah, Kurdistan Region, Iraq; 2 Medical Doctor (MD), School of Medicine, Ahvaz Jondishapour University of Medical Sciences, Ahvaz, Iran; 3 Department of Radiologic Technology, Faculty of Paramedicine, Ahvaz Jundishapur University of Medical Sciences, Ahvaz, Iran; 4 School of Biomedical Engineering, ShanghaiTech University, Shanghai, 201210 China; 5 School of Medicine, Tabriz University of Medical Sciences, Tabriz, Iran; 6 Research Center for Molecular and Cellular Imaging, Tehran University of Medical Sciences, Tehran, Iran

**Keywords:** Bone diseases, Metabolic, Osteoporosis, Lower extremity, Hip, Artificial intelligence, Machine learning, Meta-analysis

## Abstract

**Background:**

Osteoporosis is a significant health problem in the skeletal system, associated with bone tissue changes and its strength. Machine Learning (ML), on the other hand, has been accompanied by improvements in recent years and has been in the spotlight. This study is designed to investigate the Diagnostic Test Accuracy (DTA) of ML to detect osteoporosis through the hip dual-energy X-ray absorptiometry (DXA) images.

**Methods:**

The ISI Web of Science, PubMed, Scopus, Cochrane Library, IEEE Xplore Digital Library, CINAHL, Science Direct, PROSPERO, and EMBASE were systematically searched until June 2023 for studies that tested the diagnostic precision of ML model-assisted for predicting an osteoporosis diagnosis.

**Results:**

The pooled sensitivity of univariate analysis of seven studies was 0.844 (95% CI 0.791 to 0.885, *I*^2^ = 94% for 7 studies). The pooled specificity of univariate analysis was 0.781 (95% CI 0.732 to 0.824, *I*^2^ = 98% for 7 studies). The pooled diagnostic odds ratio (DOR) was 18.91 (95% CI 14.22 to 25.14, *I*^2^ = 93% for 7 studies). The pooled mean positive likelihood ratio (LR^+^) and the negative likelihood ratio (LR^−^) were 3.7 and 0.22, respectively. Also, the summary receiver operating characteristics (sROC) of the bivariate model has an AUC of 0.878.

**Conclusion:**

Osteoporosis can be diagnosed by ML with acceptable accuracy, and hip fracture prediction was improved via training in an Architecture Learning Network (ALN).

**Supplementary Information:**

The online version contains supplementary material available at 10.1186/s12938-023-01132-9.

## Background

Osteoporosis is one of the major health problems in the skeletal system, which is associated with changes in bone tissue and its strength in a way that will be prone to fracture [1]. This disease is prevalent and can strike people of all nationalities with many older men and women [2]. Many factors increase the risk of osteoporotic fractures, such as low peak bone mass, hormonal factors, the use of certain drugs (e.g., glucocorticoids), smoking, lack of physical activity, lack of calcium and vitamin D, race, small body size, and familial history of skeletal disorders [3].

According to World Health Organization (WHO) reports, 15.7% of men and women aged 50 years or more in 2000 in the Americas had osteoporosis [4]. About half of the women have some grade of osteopenia in the hip and neck of the femur [5]. Hip fractures are more common than any other type, in which half of Caucasian adult females will experience an osteoporotic fracture during their life [6]. A hip fracture can increase fatality by up to 15% during the 1st year, and as many as above 70% of survivors have a profound disability in doing functions [2].

Clinical evaluation of osteoporosis is challengeable and recognizes cases at higher risk of hip fracture by Bone Mineral Density (BMD) results [7]. Two factors express the BMD; the *T* score and the *Z* score [8]. This scale of bone density is commonly used as a representative for total bone strength and is indicated as grams of mineral per square centimetre or grams per cubic centimetre [9]. Access to average peak bone mass is necessary for intercepting osteoporosis. Bone mass is distinguished by dual-energy X-ray absorptiometry (DEXA, or DXA), quantitative computed tomography (CT) scan, and a peripheral ultrasound [10, 11].

Using DXA has several disadvantages, such as incidence related to measurement faults that make happen by the surrounding soft tissues, radiation exposure, and high system price [12–14]. The effortless availability of BMD examinations is essential, specifically in developing countries [15, 16].

The appropriate way to calculate data collection and extract unexpected risk factors for preventive medicine is machine learning (ML) and artificial intelligence (AI). ML is a subset of computer science attached to algorithm expansion, authorizing the computer to learn from examples [17, 18]. In recent years ML is the new method in medical divination models that have transpired specifically in osteoporotic. Few ML studies predict osteoporotic fracture and improve hip fracture prediction beyond logistic regression (LR) [19]. ML algorithms have been used to indicate the chance of hip fractures, length of rehabilitation, patient resource utilization after lumbar spinal fusion, and length of aftercare for hip fracture patients [20–23].

ML may help distinguish osteoporosis risk, grading, and conclusion; a complex of clinical, laboratory, and DXA variables show positive results [24, 25]. Diagnostic Test Accuracy (DTA) research can investigate the role of ML in osteoporosis diagnosing.

## Results

### Study selection and characteristics

**Fig. 1:**
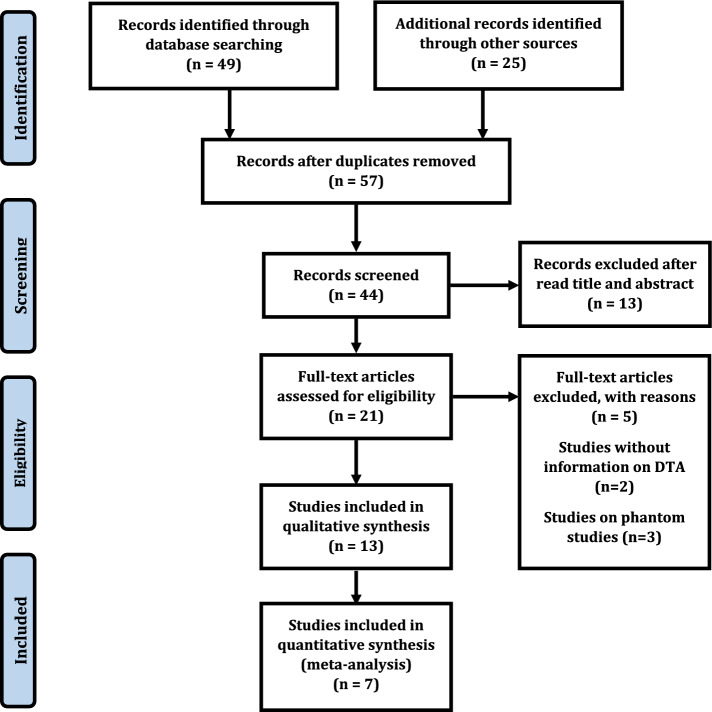
Study flow diagram showing how to extract articles

Finally, 57 studies were included in the primary search, and seven duplicate studies were removed. Thirteen retrospective studies were included after the title, abstract, and full paper screening; then, seven studies were included for analysis. Six studies were excluded because no diagnostic accuracy was reported (Fig. 1) [15, 19, 20, 24, 26–34]. The mean age of people was 73.84 ± 8.45 years, and 159,644 (50.75%) were female (Table 1). The ML algorithms were classified into an artificial neural network (ANN), Support Vector Machine (SVM), Random Forest (RF), k-nearest neighbors (KNN), Logistic Regression (LR), RSF, AdaBoost, CatBoost, ExtraTrees, XGBoost, Deep-TEN, ResNet-18, RUSBoost, Superlearner, XGBoost, NN, Decision Trees (DT), CNN; ResNet18, ResNet34, GoogleNet, EcientNet b3, EcientNet b4, XGB, BFDA, CB, LR, bagFDA, xgbTree [15, 19, 20, 24, 26–34]. Finally, all included studies used ML with ANN, SVM, RF, KNN, LR, and DT algorithms (Table 1) [15, 19, 28–30, 32, 33].

**Table 1:** Summary of findings

ID	Expert radiologists involved as a control	Mean age, years	Gender, *N* (%)		AI model	Reference standard	Outcomes
			Male	Female			
Ou Yang et al. (2021)/Taiwan [33]	Yes	81.4 ± 6.95	3053	2929	ANN, SVM, RF, KNN, LR	DXA	Machine learning algorithms improve the performance of screening for osteoporosis
de Vries et al. (2021)/The Netherlands [26]	Yes	> 50	2564	5014	ANN, RSF	DXA	Major Osteoporotic Fracture can be done with adequate discriminative performance
Shtar et al. (2021)/Israel [20]	Yes	83.1 ± 7.4	514	1382	AdaBoost, CatBoost, ExtraTrees, KNN, RF, SVM, XGBoost	DXA	hip fracture patients are superior to linear and logistic regression models
Kuo et al., (2020)/China [32]	Yes	66.1 ± 1.7	18	151	Deep-TEN, ResNet-18	DXA	The bone texture model can detect osteoporosis and predict the FRAX score
Engels et al., (2020)/Germany [27]	Yes	75.67 ± 6.20	147,377	140,709	LR, SVM, RF, RUSBoost, Superlearner, XGBoost	DXA	Super learners showed poorer discrimination and calibration in the validation set
Villamor et al., (2020)/Spain [34]	Yes	81.4 ± 6.95	NA	137	SVM, LR, NN, RF	DXA	Prediction of the hip fracture without interrupting the actual clinical workflow
Galassi et al., (2020)/Spain [28]	Yes	81.4 ± 6.95	NA	137	LR, SVM, DT, RF	DXA	Clinical, geometric, and biomechanical variables from the finite element simulation of a side fall are used as independent variables to train the models
amamoto et al. (2020)/Japan [15]	Yes	82.7 ± 8.3	346	877	CNN; ResNet18, ResNet34, GoogleNet, E cientNet b3, E cientNet b4	DXA	High accuracy for the CNN models diagnosed osteoporosis from hip radiographs
Erjiang et al., (2020)/China [24]	Yes	60.24 ± 10.56	107	1162	XGB, BFDA, NN, CB, LR, RF, SVM	DXA	MLTs could improve DXA detection of osteoporosis classification in older men and women
Kong et al., (2020)/Republic of Korea [31]	Yes	61.2 ± 8.7	970	1257	CB, SVM, LR	DXA	CatBoost model, the top predicting factors
Hussain et al., (2019)/Republic of Korea [30]	Yes	NA	150		RF	DXA	RF will reduce workload and improve the use of X-ray devices
Ho-Le et al., (2017) [29]/Australia	Yes	69.1 ± 6.4	NA	1167	ANN, LR, KNN, SVM	DXA	ANNs can predict hip fractures
Kruse et al., (2017) [19]/Denmark	Yes	74.5 ± 65.5, 69.3 ± 59.9	717	4722	bagFDA, xgbTree	DXA	Machine learning can improve hip fracture prediction beyond logistic regression

*DXA* Dual-energy X-ray absorptiometry, *Deep-TEN* Deep Texture Encoding Network, *ResNet-18* three blocks of Residual Network with 18 layers, *SVM* Support Vector Machine with *RBF* radial basis function, *LR* Logistic Regression, *SNN* Shallow Neural Networks, *RF* Random Forest, convolutional neural network (CNN), Decision Trees (DT), eXtreme Gradient Boosting (XGB), *BFDA* Bagged Flexible Discriminant Analysis, *CB* CatBoost, *ANN* artificial neural network, *bagFDA* bootstrap aggregated flexible discriminant analysis model, *xgbTree* eXtreme Gradient Boosting, *RSF* Random Survival Forests, *GB* Gradient boosting, *KNN* k-nearest neighbors algorithm

### Risk of bias within studies

**Fig. 2:**
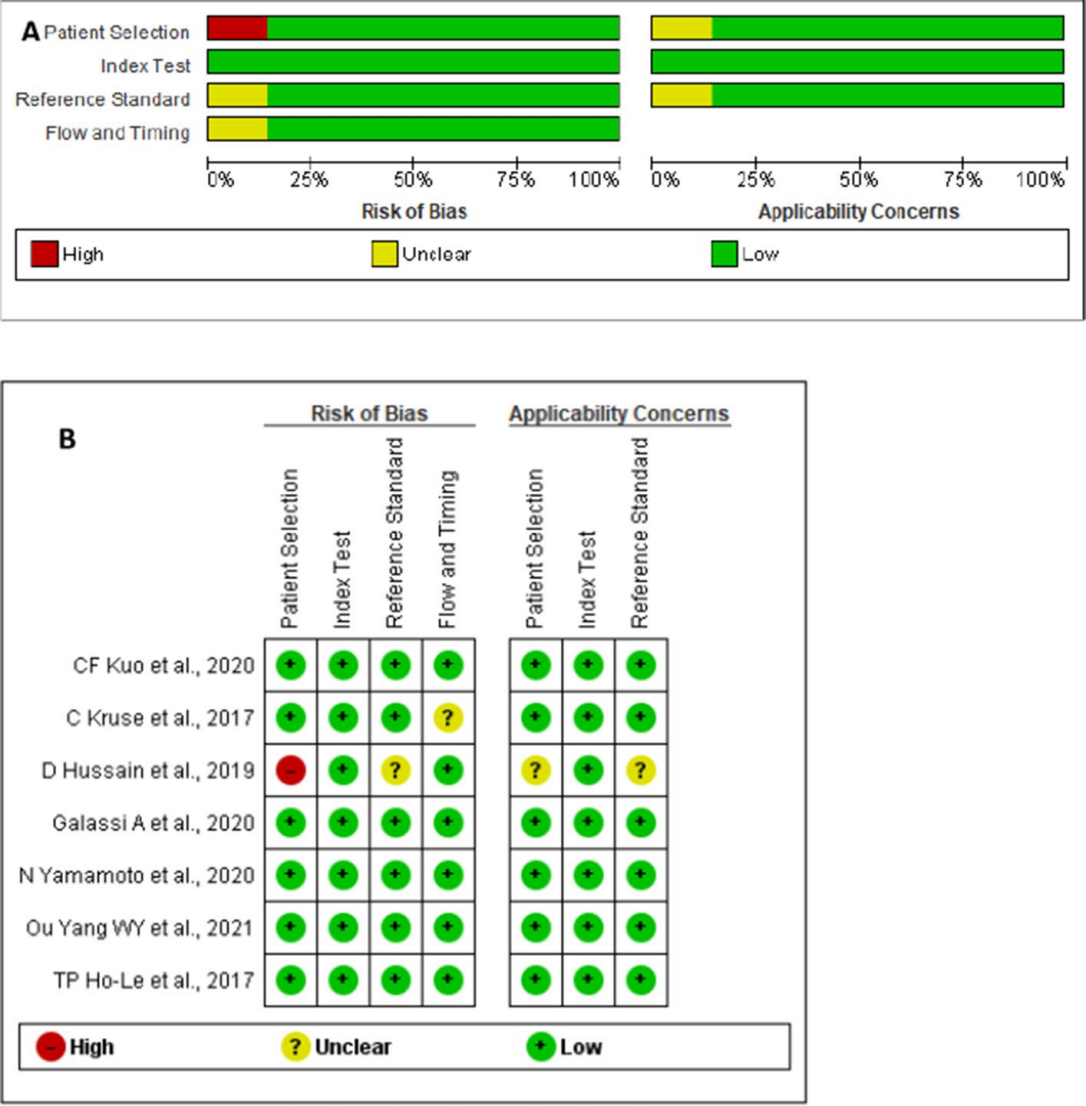
**A** Risk of bias and applicability concerns graph; review authors’ judgments about each domain presented as percentages across included studies. **B** Risk of bias and applicability concerns summary; review authors' judgments about each domain for each included study

**Fig. 3:**
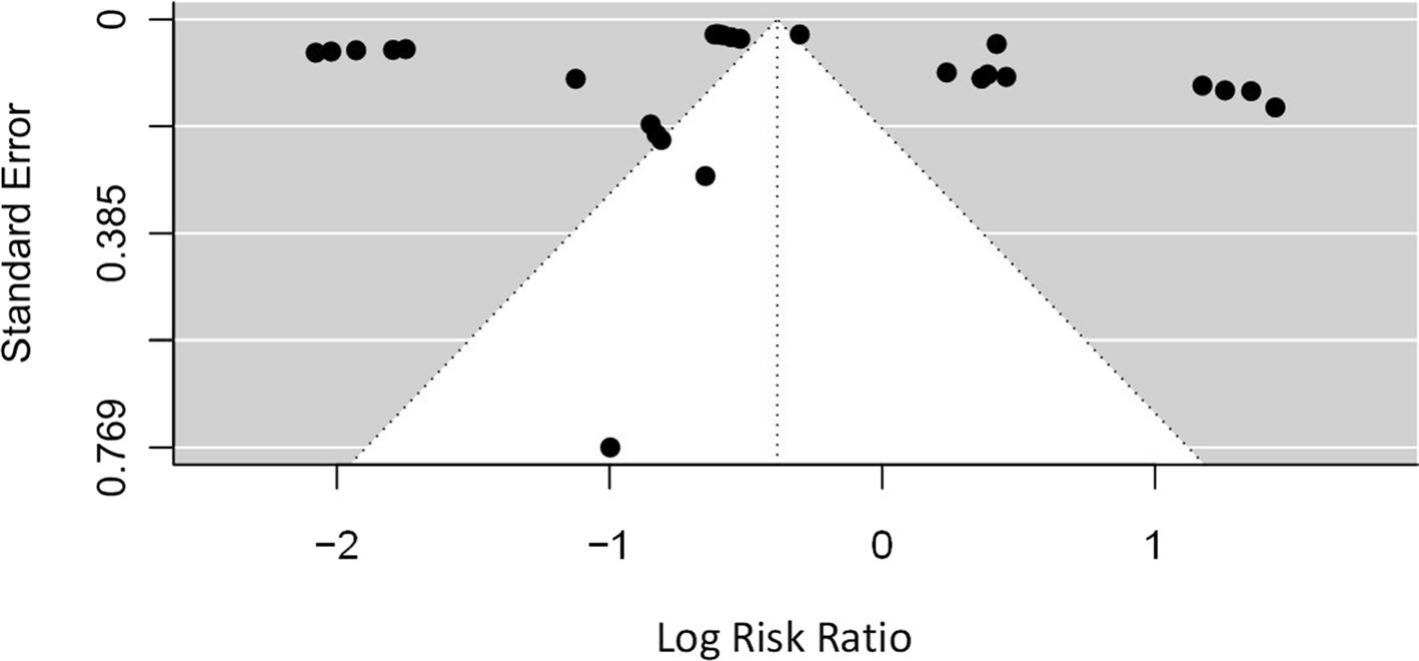
Funnel plot showing the low likelihood of publication bias in all included studies

The QUADAS-2 criteria assess the validity of included research and the possibility of bias (Fig. 2). There were no studies with high-risk biases. The points will be symmetrically distributed around the true effect in the shape of an inverted funnel when publication bias is very low, as shown in Fig. 3.

### Diagnostic test accuracy (DTA) of all included studies

**Fig. 4:**
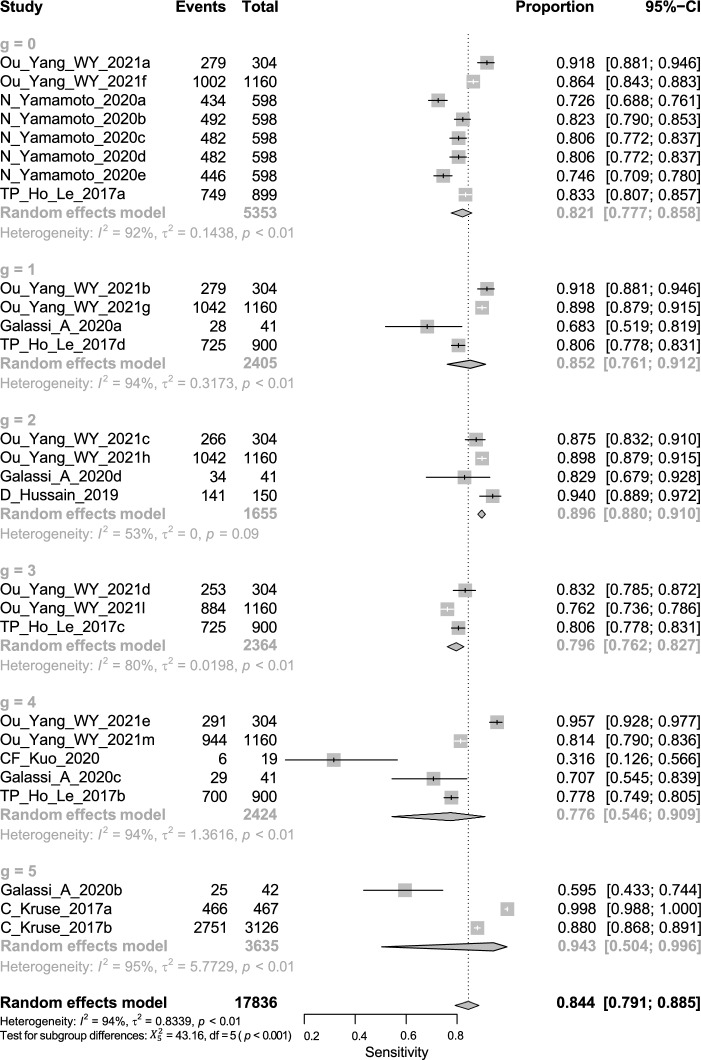
Univariate sub-group analysis of sensitivity with random model based on Network Architecture. G represents sub-group analysis of data, when g = 0 (ANN), g = 1 (SVM), g = 2 (RF), g = 3 (KNN), g = 4 (LR), and g = 5 (DT)

**Fig. 5:**
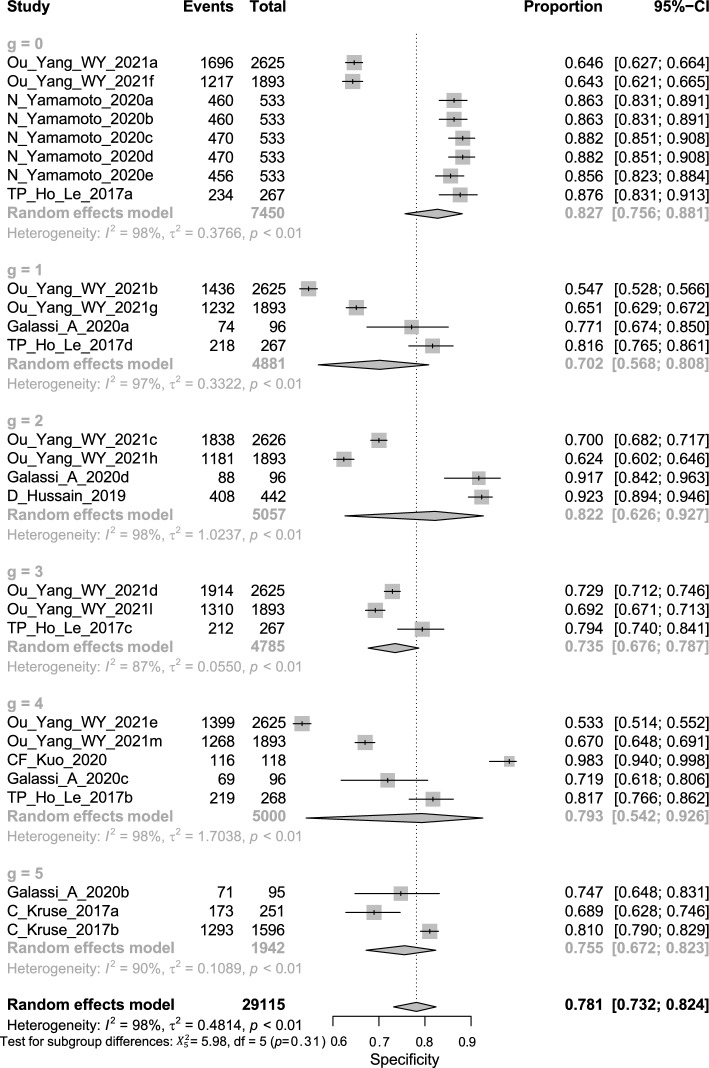
Univariate sub-group analysis of specificity with random model based on Network Architecture. G represents sub-group analysis of data, when g = 0 (ANN), g = 1 (SVM), g = 2 (RF), g = 3 (KNN), g = 4 (LR), and g = 5 (DT)

**Fig. 6:**
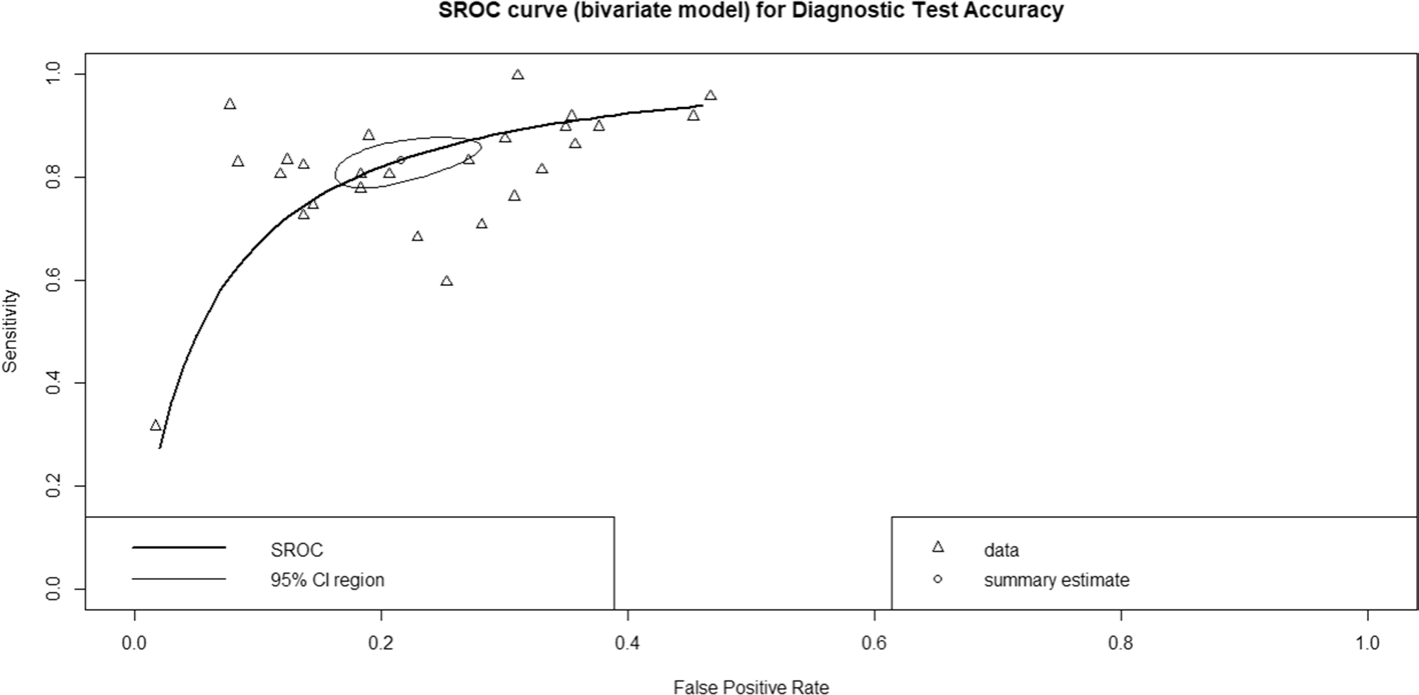
Univariate sub-group analysis of DOR with random model based on Network Architecture. G represents sub-group analysis of data, when g = 0 (ANN), g = 1 (SVM), g = 2 (RF), g = 3 (KNN), g = 4 (LR), and g = 5 (DT)

Overall, this is of all DTA; the pooled sensitivity of univariate analysis of seven studies was 0.844 (95% CI 0.791 to 0.885, *I*^2^ = 94% for seven studies) shown in Fig. 4. The pooled specificity of univariate analysis was 0.781 (95% CI 0.732 to 0.824, *I*^2^ = 98% for 7 studies) as shown in Fig. 5. The pooled diagnostic odds ratio (DOR) was 18.91 (95% CI 14.22 to 25.14, *I*^2^ = 93% for 7 studies) as shown in Fig. 6. The positive likelihood ratio (LR^+^) ranges from 3.23 to 4.25 with pooled mean of 3.7 (Table 2); likewise, the negative likelihood ratio (LR^−^) spans from 0.19 to 0.26 with pooled mean of 0.22. The SROC of the bivariate model has an AUC of 0.878 (Additional file 1: Fig. S1).

**Table 2:** DTA estimated from all included studies using the (2 × 2) truth table

Amount	LR^+^	LR^−^	Accuracy	Precision	F1 score
Minimum	3.23	0.19	0.6975	0.589	0.6783
Maximum	4.25	0.26	0.78	0.6448	0.7425
Average	3.7	0.22	0.75	0.62	0.714

The accuracy of all included studies ranges from 0.6975 to 0.78 with a mean of 0.75 (Table 2), while the precision ranges from 0.589 to 0.6448 with a mean of 0.62 (Table 2), the F1 score has a mean of 0.714 and ranges from 0.6783 to 0.7425 (Table 2).

### DTA based on algorithms architecture

The Algorithms Architecture analysis was divided into ANN, SVM, RF, KNN, LR, and DT. These results reveal a significant difference in the sensitivity of the categories of Algorithms architecture (*P* value = 0.0028) Fig. 4. These results show no significant difference in the specificity of the categories of Algorithms architecture (*P* value = 0.3086) Fig. 5. These results indicate no significant difference in the DOR of the categories of Algorithms architecture (*P* value = 0.0843) Fig. 6.

### DTA based on gender

The DTA analysis was divided into two subgroups, male and female. These results show no significant difference in the sensitivity of the categories of gender (*P* value = 0.3275) (Additional file 1: Fig. S2). These results indicate a significant difference in the specificity of the categories of gender (*P* value = 0.0226) (Additional file 1: Fig. S3). These results show no significant difference in the DOR of the categories of gender (*P* value = 0.301) (Additional file 1: Fig. S4).

### DTA based on continent

The DTA analysis was divided into Asia, Europe, and Australia. These results show a significant difference in the sensitivity of the Continent categories (*P* value = 0.0099) (Additional file 1: Fig. S5). These results indicate no significant difference in the specificity of the Continent categories (*P* value = 0.3439) (Additional file 1: Fig. S6). These results show no significant difference in the DOR of the Continent categories (*P* value = 0.6027) (Additional file 1: Fig. S7).

## Discussion

In recent years, the results obtained using AI in detecting bone fractures and osteoporosis have been promising [18, 35]. In this current study, ML has been used in pelvic DXA images of patients prone to osteoporosis with different architecture models. The resulting pooled sensitivity, specificity, DOR, AUC, accuracy, and precision were 0.844 (95% CI 0.885, *I*^2^ = 94%), 0.781 (95% CI 0.732 to 0.824, *I*^2^ = 98%), 18.91 (95% CI 14.22 to 25.14, *I*^2^ = 93%), 0.878, 0.75 (ranges from 0.6975 to 0.78), and 0.62 (ranges from 0.589 to 0.6448) respectively, and also pooled LR^+^, LR^−^, and F1 score were 3.7 (ranges from 3.23 to 4.25), 0.22 (ranges from 0.19 to 0.26), 0.714 (ranges from 0.6783 to 0.7425) respectively.

According to evidence, this is the first study which analyzed the DTA of ML algorithms for detecting osteoporosis by assessing X-Ray hip bone; therefore, this study can be used as an indicator for comparing with other study results.

Suitable machine learning is defined by high accuracy factors such as AUC, sensitivity, and specificity, which can correctly classify suspects from disease and non-disease. This meta-analysis reported a pooled AUC of 0.878, a high result for this study. ML algorithms improve the performance of screening for osteoporosis without interrupting the actual clinical workflow [33, 34], and major osteoporotic fractures can be done with adequate discriminative performance [26]. The AUC result reported ranges from 0.663 to 0.92 [15, 24, 29, 31]; however other studies were not reported the AUC result.

To interpret the results, a DOR of 18.91 (95% CI 14.22 to 25.14, *I*^2^ = 93%) generally means that the use of ML in the diagnosis of osteoporosis is valuable. Due to the necessity of reporting the convergence of the results along with the accuracy, precision is also mentioned. Precision equal to 0.62 (ranges from 0.589 to 0.6448) indicates a relative convergence besides the accuracy of 0.75 (ranges from 0.6975 to 0.78). Based on these overall results, ML can be diagnosed with osteoporosis from non-osteoporosis. Also, likelihood ratios are important factors which could help to improve clinical judgment and shows the range of disease frequencies, and LR^+^ greater than 10 produces a greater pretest probability. The LR^−^ less than 0.1 produces conclusive changes in the post-test probability [36]. The pooled positive LR^+^ and LR^−^ are 3.7 (ranges from 3.23 to 4.25) and 0.22 (ranges from 0.19 to 0.26), respectively. The pooled LR^+^ of 3.7 simply means that diagnosis of osteoporosis through the hip DXA images is 3.7 times more likely to be diagnosed while ML is used; likewise, the pooled LR^−^ of 0.22 means osteoporosis has a higher likelihood of a negative test for the ML algorithm than non-osteoporosis.

The pooled F1 score of this study was 0.714. The F1 score is a numerical score between 0 and 1, and the closer this number is to 1, the more valuable the method studied [37]. This score results from the average weight of recall and precision, which has a significant place in data interpretation. It can be reduced the number of false negatives and positives.

The sub-group analysis based on the ML architecture and gender was done to assess these factors' influence on the DTA results. The Algorithms architecture analysis results showed a significant difference in the pooled sensitivity of the categories of Algorithms architecture (*P* value = 0.0028); thus, DT architecture has higher pooled sensitivity than other architectures 0.943 (95% CI 0.504 to 0.996, *I*^2^ = 95%) and pooled LR architecture has lower sensitivity than other architectures 0.776 (95% CI 0.546 to 0.909, *I*^2^ = 94%). In contrast, pooled specificity, DOR, AUC, accuracy, and precision were not statistically significant between Algorithms architectures; however, these algorithms have high results which can be used in future studies. On the other hand, there was a significant difference in the pooled specificity of the categories of gender (*P* value = 0.0226); thus, the female has higher pooled specificity than the male 0.77 (95% CI 0.679 to 0.842, *I*^2^ = 99%) and the male has lower pooled specificity than female 0.659 (95% CI 0.639 to 0.68, *I*^2^ = 17%). Also, there was a significant difference in the pooled sensitivity of the categories of Continent (*P* value = 0.0099); thus, Europe has higher pooled sensitivity than others 0.88 (95% CI 0.836 to 0.913, *I*^2^ = 81%).

Variation in the type of datasets used (single-centre or multicenter) leads to differences in the resulting data; thus, single-centre datasets seem to have less heterogeneity; however, another factor that causes heterogeneity in the studies included in the analysis was the geographical dispersion [38]. Also, studies included were from different countries (Taiwan, China, Spain, Japan, the Republic of Korea, Australia, and Denmark) with different geographic locations, which could be a source of potential heterogeneity. Consequently, the continent (Asia, Europe, and Australia) was analyzed as a subgroup.

Different architectures in ML models and the age difference among participants in the included studies in this meta-analysis are counted as other possible causes of heterogeneity. For instance, the results obtained by using regions of random forest in phantom and human models and comparing the results with each other showed an accuracy of 0.962 and 0.988 [30]. However, the calculated accuracy in this current meta-analysis was 0.75, which is lower than the more homogeneous studies included in the analysis. In addition to the possible causes of heterogeneity, retrospective studies were the most critical limitation of this study, so the design of prospective studies in this field can significantly contribute to future progression. Another limitation of the study is the sample size considered for the sub-group analysis for continents is too small, this may not generalize or fully represent results from each continent under consideration.

## Conclusion

This meta-analysis on DTA of ML algorithms for detecting Osteoporosis by assessing Hip Bone shows the ML has an acceptable performance to diagnose Osteoporosis. Hip fracture prediction was improved via training in an Architecture Learning Network. However, further studies with greater homogeneity are needed to draw more accurate conclusions about the results of DTA of ML in osteoporosis.

## Methods

### Protocol and registration

This meta-analysis study was reported according to Preferred Reporting Items for Systematic Reviews-Diagnostic Test Accuracy (PRISMA-DTA) and Meta-analysis Of Observational Studies in Epidemiology (MOOSE) guidelines.

### Eligibility criteria

A bone mineral density at the femoral neck equal to or less than 2.5 standard deviations (SDs) below the mean for a young person of the same sex is diagnostic of osteoporosis. At study entry, bone mineral density (BMD; g/cm^2^) was measured at the lumbar spine and femoral neck. The measurement was done with the DXA based on the femoral neck BMD; the femoral neck BMD T-score was calculated as the number of SD was different from the young normal level (ideal or peak bone mineral density).

### Information sources

The ISI Web of Science, PubMed, Scopus, Cochrane Library, IEEE Xplore Digital Library, CINAHL, Science Direct, PROSPERO, and EMBASE were systematically searched until June 2023 for studies that tested the diagnostic precision of ML model-assisted for predicting an osteoporosis diagnosis.

### Search

One experienced librarian [K⋅SH] drafted search strategies and refined them through team discussion. The search used medical subject headings (MeSh) terms including “Deep Learning”, “Machine Learning”, “Artificial Intelligence”, “Bone Mineral Density”, “BMD”, “Fracture Risk Assessment Tool”, ” Lower Extremity”, “Hip” in different combinations. Papers that did not fit into the study’s conceptual framework were excluded.

### Summary measures

The principal outcome of interest was diagnostic accuracy = ((TP + TN)/(TP + FN + FP + TN)), sensitivity = TP/(TP + FN), specificity = TN/(FP + TN), precision = (TP/TP + FP), F1 Score = 2 X (Precision X Recall/Precision + Recall), positive likelihood ratio (LR^+^) = (sensitivity/1 − specificity), negative likelihood ratio (LR^−^) = (1 − sensitivity/specificity), diagnostic odds ratios (DOR) = (LR^+^/ LR^−^), and the AUC of ML on detecting osteoporosis in the patients, osteoporosis versus healthy controls (HCs) [39, 40]. The secondary outcomes were to compare the accuracy, sensitivity, and specificity of the ML with BMD modalities.

### Risk of bias across studies

In terms of meta-analysis data retrieval for predicting osteoporosis in patients, osteoporosis versus healthy controls (HCs) that were true positive (TP, true osteoporosis, predicted to be osteoporosis), true negative (TN, non-osteoporosis predicted to be non-osteoporosis), false positive (FP, non-osteoporosis predicted to be osteoporosis) or false negative (FN, osteoporosis, predicted to be non- osteoporosis) were extracted.

The inclusion criteria in the original study were used to collect data for the meta-analysis on detecting osteoporosis. In addition, the year of publication, the country where the research was performed, studies method, patients’ numbers, and their ages were all recovered. The revised Quality Assessment of Diagnostic Accuracy Studies (QUADAS-2) tool was used to assess all studies’ quality and potential bias by two independent reviewers. Conflicts were settled by dialogue, and a third reviewer and reviewers assessed the first included articles independently. Two categories were evaluated: bias vulnerability and applicability within the patient selection, index test, and comparison benchmark. In the domains of flow and pacing, bias was assessed.

### Additional analyses

Using the method of DerSimonian Laird's Random Effects Model (RE), a univariate meta-analysis was performed individually for sensitivity and specificity to estimate the diagnostic accuracy of each modality [41]. The RE model was chosen because of the suspected high proportion of heterogeneity. The primary endpoints were sensitivity, specificity, a summary of receiver operating characteristics (SROC) curve, and diagnostic odds ratio (DOR). Point estimates and 95% confidence intervals (CIs) for each study were calculated to ensure consistency of sensitivity and specificity. A bivariate meta-analysis of sensitivity and specificity used R version 4.1.2 (R Foundation for Statistics Computing, Vienna, Austria, 2021) and RStudio version 1.4.1717 to obtain the SROC curve. This includes the R package. "mada" and "meta" have been implemented. Then the average AUC of SROC was estimated [42, 43]. Secondary results included the positive and negative likelihood ratio, accuracy, and F1 score. Cochran's Q test and *I*^2^ statistics assessed statistical heterogeneity between studies. For Q statistics, values in the range of 0–40% mean non-significant non-uniformity, 30–60% mean moderate non-uniformity and 75–100% mean significant non-uniformity. Means sex. Publication bias was evaluated and visualized by creating a funnel chart [32]. All *p* values are based on two-sided tests, and *p *values ≤ 0.05 were considered statistically significant. Subgroup analysis was performed by screening based on machine learning algorithms, gender, and Continent. Bias cross-study risk and applicability concern charts were evaluated using the Cochrane Review Manager version 5.4 (RevMan 5.4) software.

## Supplementary Information

**Additional file 1:** **Figure S1.** The SROC of the bivariate for DTA. **Figure S2. **Univariate sub-group analysis of sensitivity with random model based on gender. G represents sub-group analysis of data, when g = 0 (Female), and g = 1 (Male). **Figure S3. **Univariate sub-group analysis of specificity with random model based on gender. G represents sub-group analysis of data, when g = 0 (Female), and g = 1 (Male). **Figure S4. **Univariate sub-group analysis of DOR with random model based on gender. G represents sub-group analysis of data, when g = 0 (Female), and g = 1 (Male). **Figure S5. **Univariate sub-group analysis of sensitivity with random model based on gender. G represents sub-group analysis of data, when g = 0 (Asia), g = 1 (Europe), and g = 2 (Australia). **Figure S6. **Univariate sub-group analysis of specificity with random model based on gender. G represents sub-group analysis of data, when g = 0 (Asia), g = 1 (Europe), and g = 2 (Australia). **Figure S7. **Univariate sub-group analysis of DOR with random model based on gender. G represents sub-group analysis of data, when g = 0 (Asia), g = 1 (Europe), and g = 2 (Australia).

## Data Availability

Not applicable.
