# Supplementary material for: Machine learning algorithms for diagnosis of hip bone osteoporosis: a systematic review and meta-analysis study
Source: Biomed Eng Online. 2023 Jul 10;22:68. doi: 10.1186/s12938-023-01132-9 (PMC10331995; doi:10.1186/s12938-023-01132-9)

**Supplementary Figure 1.** The SROC of the bivariate for DTA.


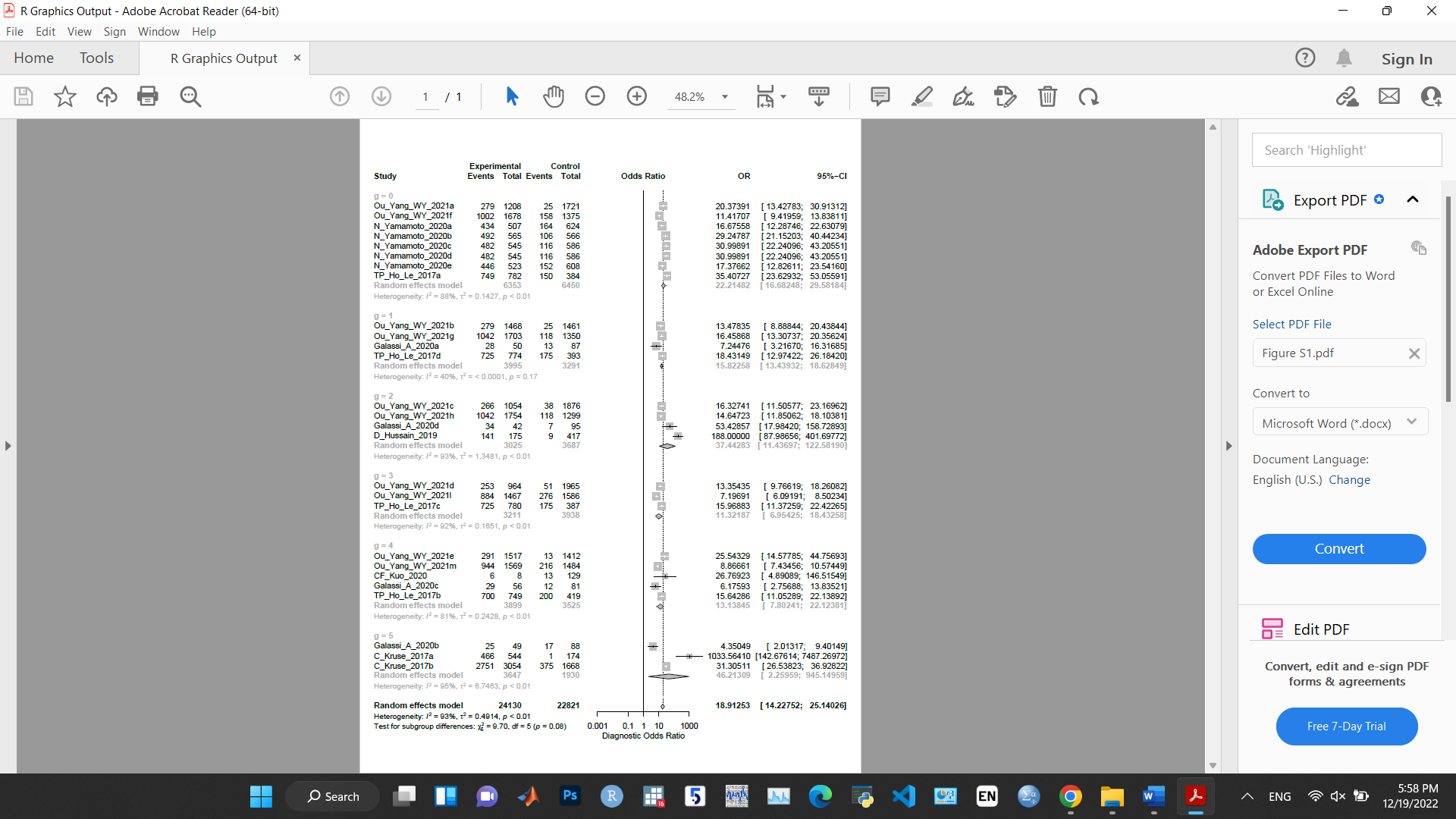


**Supplementary Figure 2.** Univariate sub-group analysis of sensitivity with random model based on gender. G represents sub-group analysis of data, when g = 0 (Female), and g = 1 (Male).


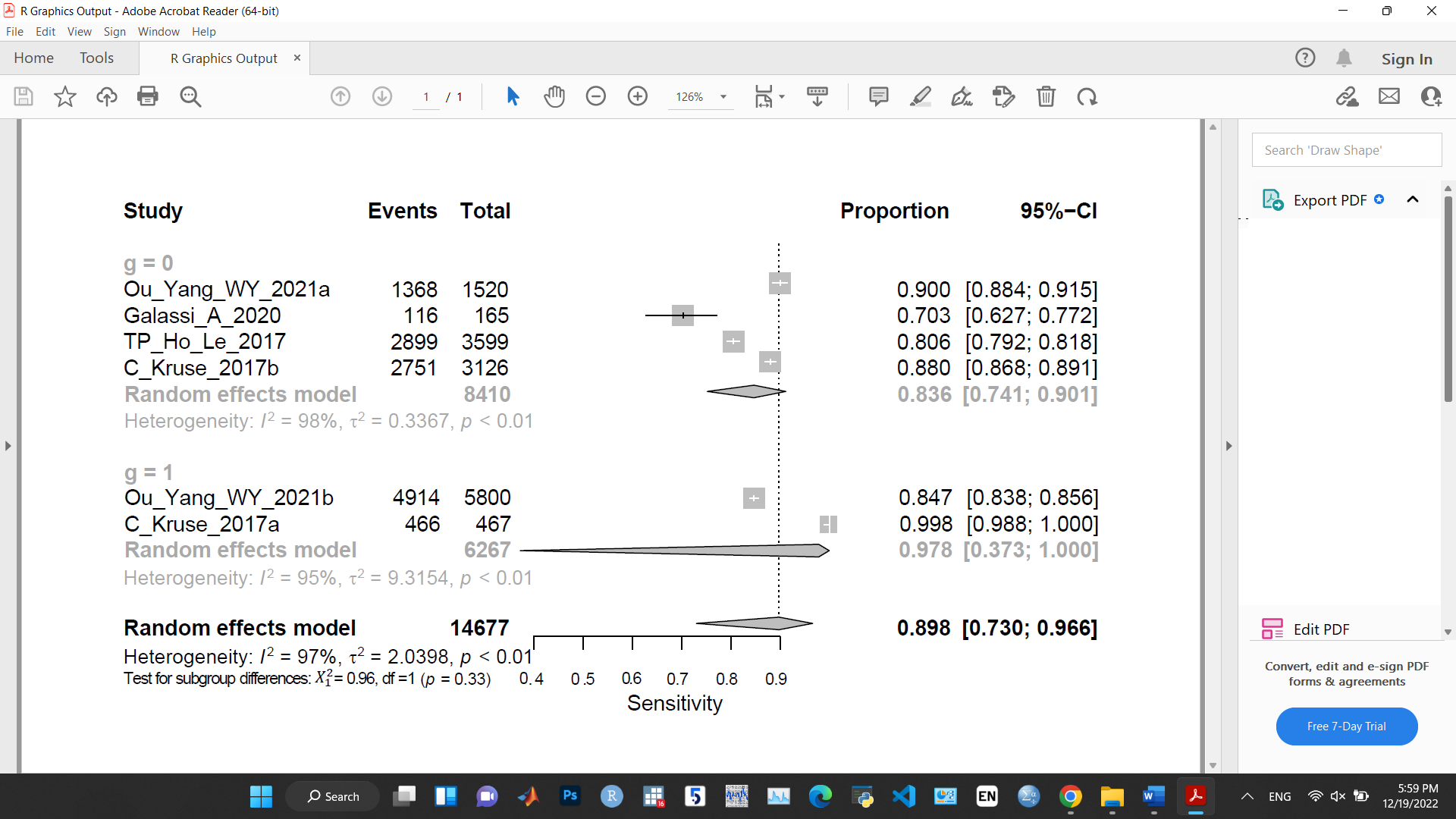


**Supplementary Figure 3.** Univariate sub-group analysis of specificity with random model based on gender. G represents sub-group analysis of data, when g = 0 (Female), and g = 1 (Male).


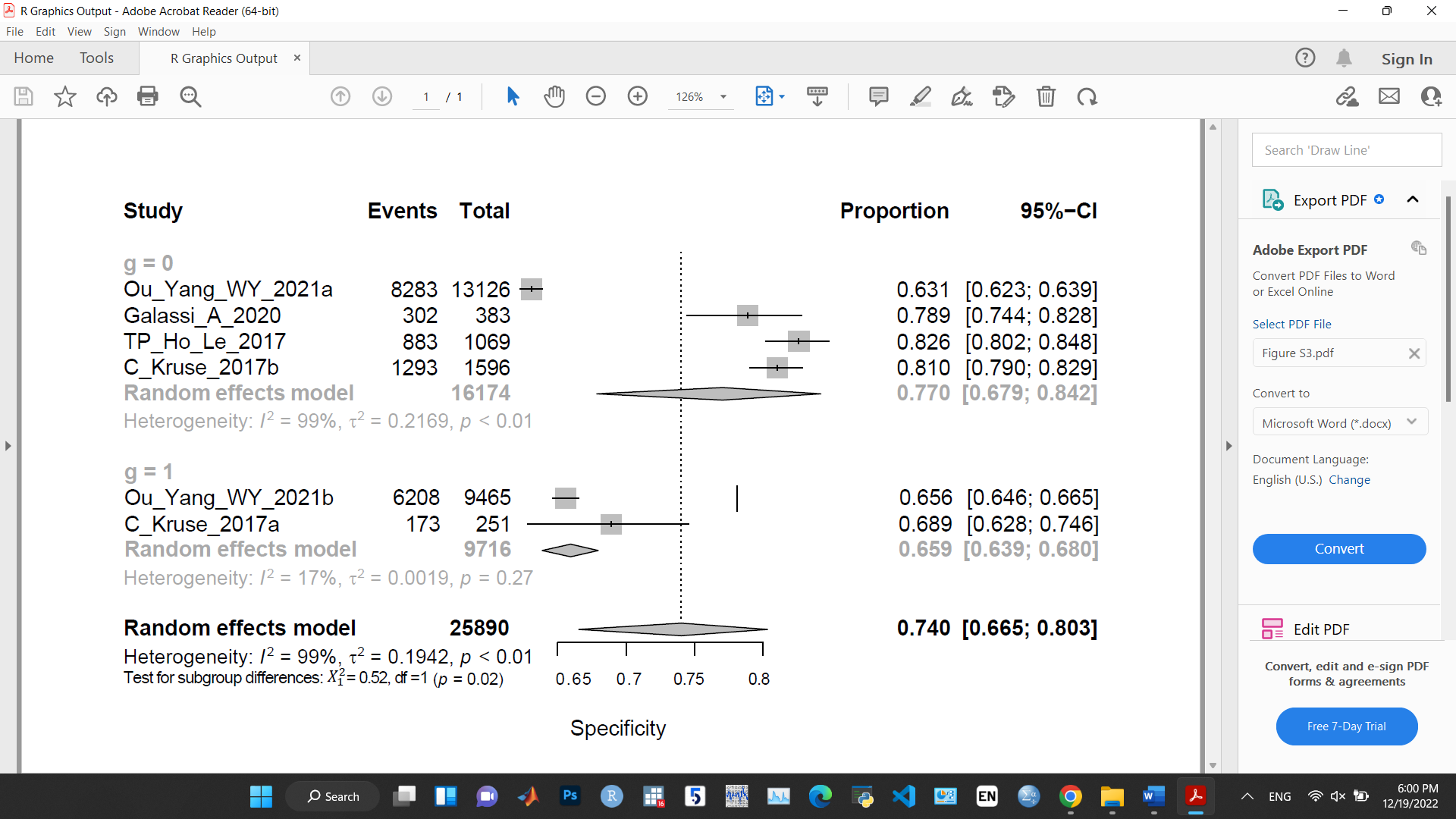


**Supplementary Figure 4.** Univariate sub-group analysis of DOR with random model based on gender. G represents sub-group analysis of data, when g = 0 (Female), and g = 1 (Male).


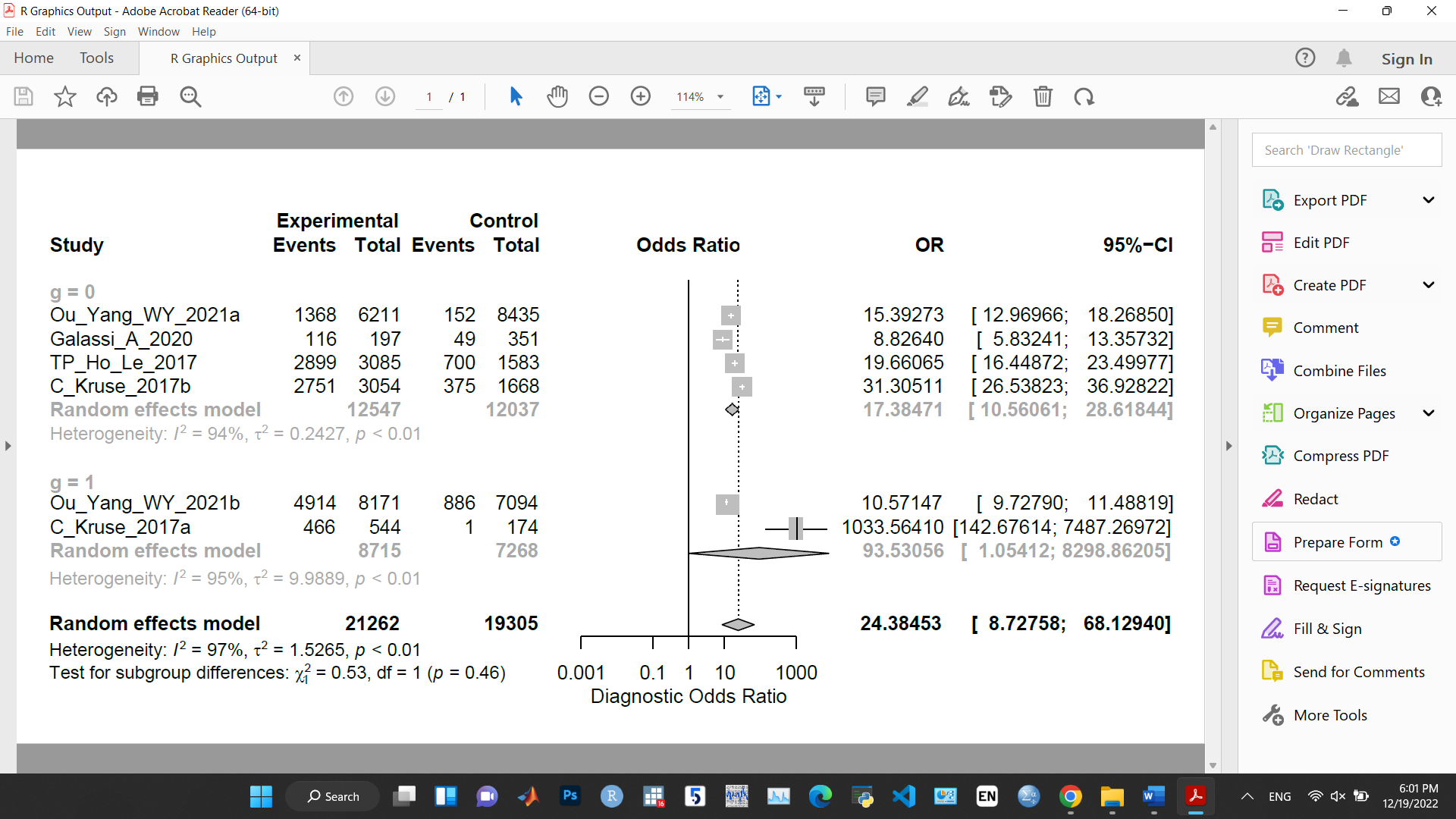


**Supplementary Figure 5.** Univariate sub-group analysis of sensitivity with random model based on gender. G represents sub-group analysis of data, when g = 0 (Asia), g = 1 (Europe), and g = 2 (Australia).


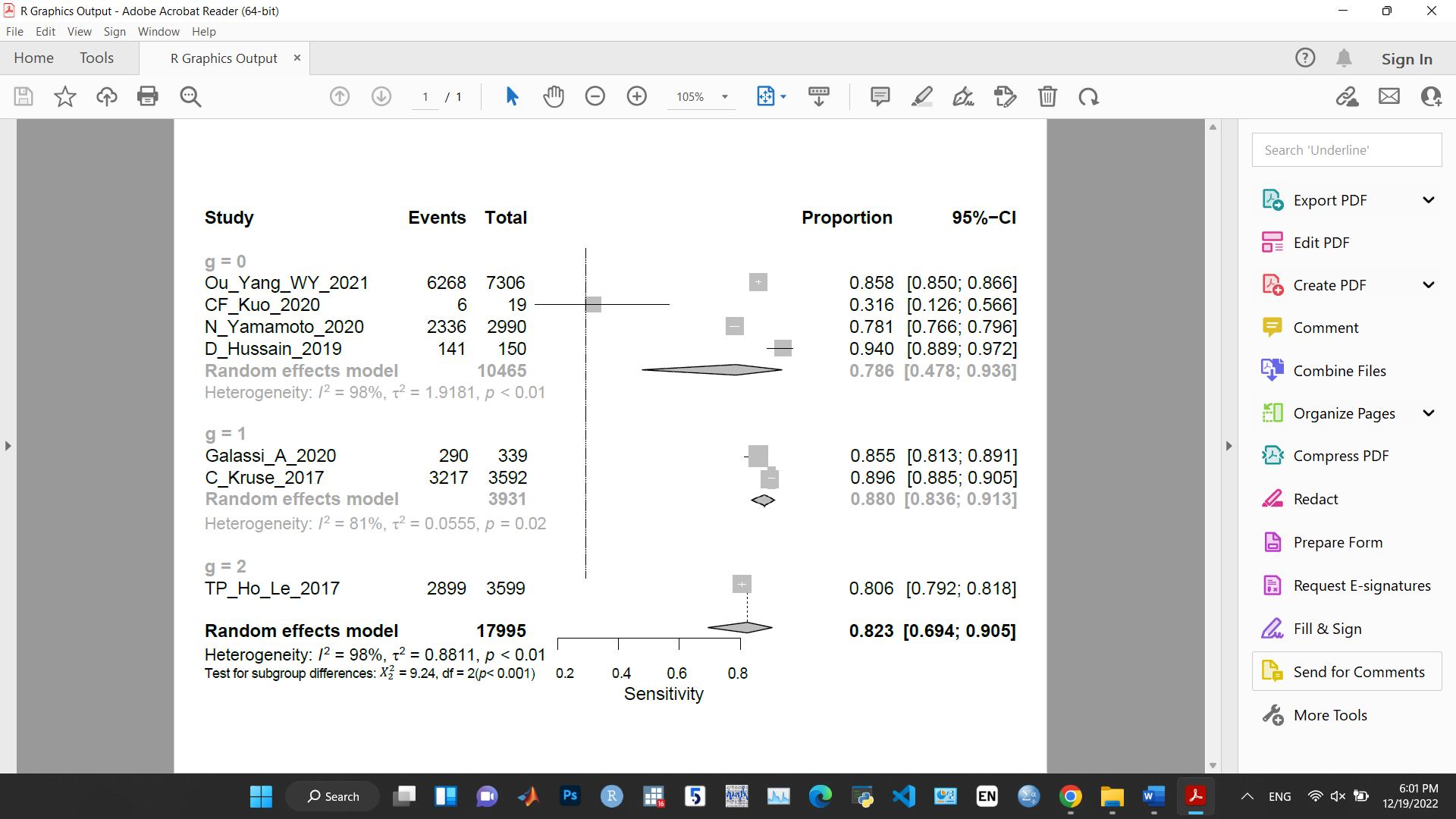


**Supplementary Figure 6.** Univariate sub-group analysis of specificity with random model based on gender. G represents sub-group analysis of data, when g = 0 (Asia), g = 1 (Europe), and g = 2 (Australia).


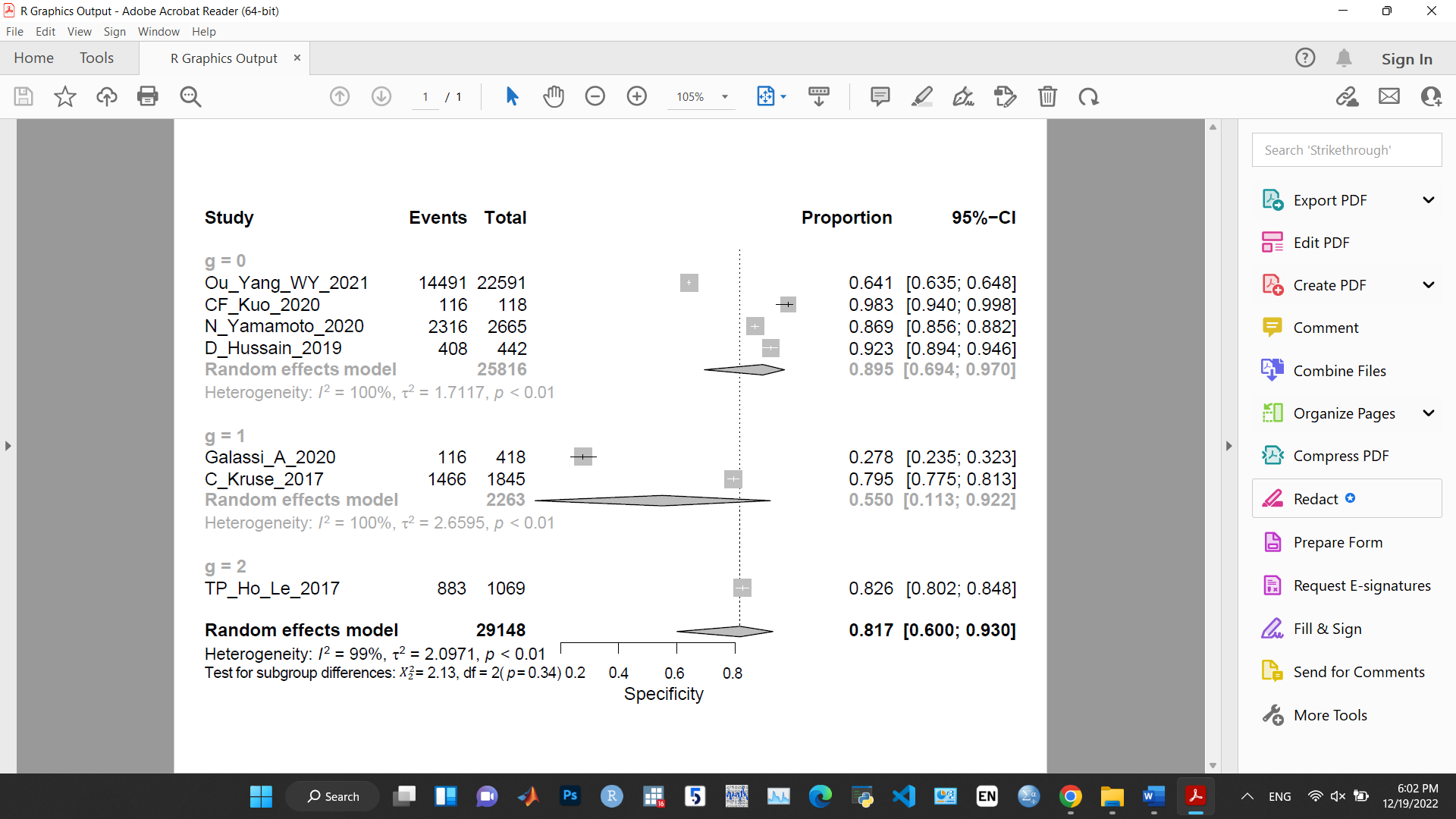


**Supplementary Figure 7.** Univariate sub-group analysis of DOR with random model based on gender. G represents sub-group analysis of data, when g = 0 (Asia), g = 1 (Europe), and g = 2 (Australia).


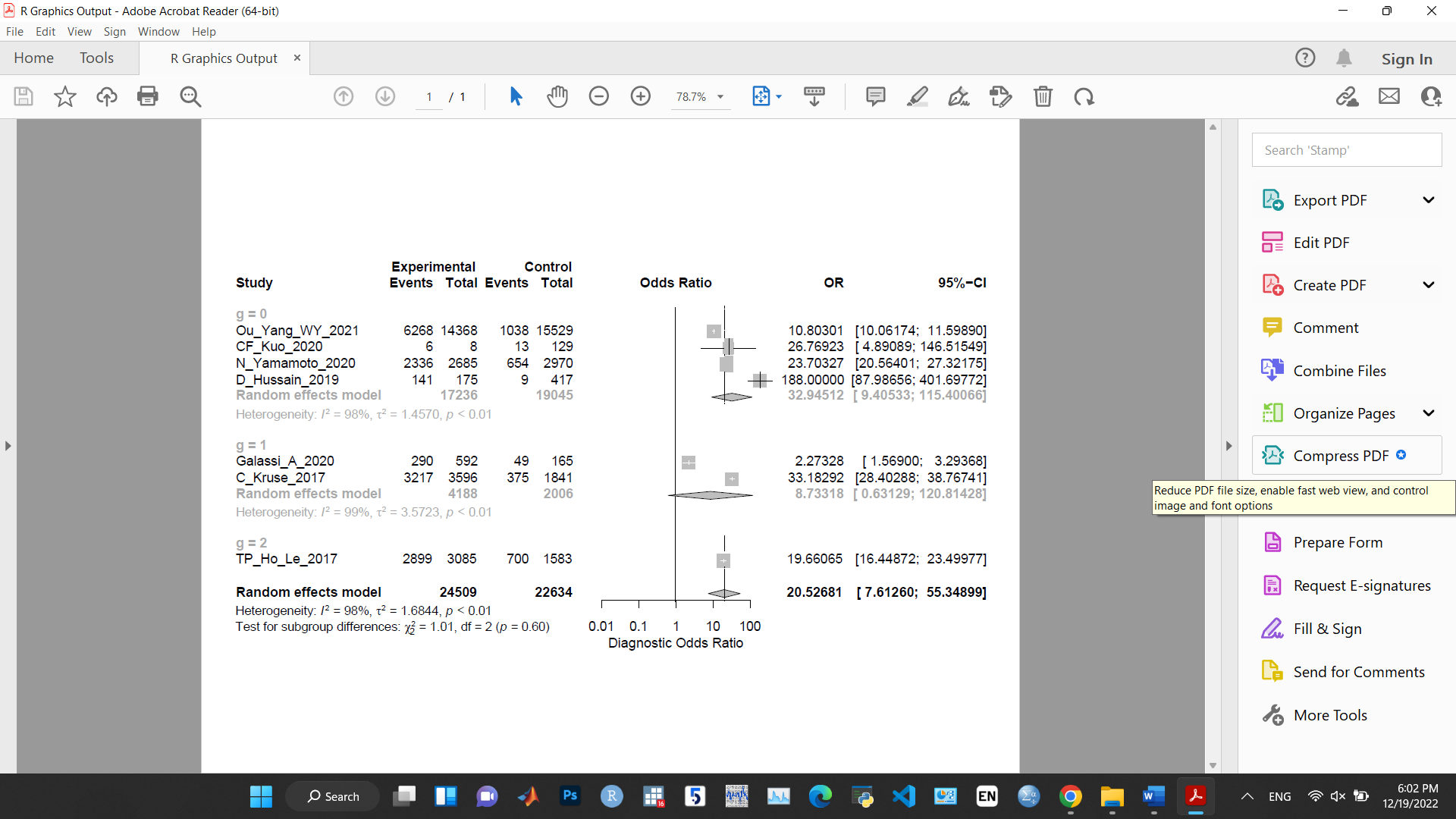

Supplement: Supplementary file 1 — Additional file 1: Figure S1. The SROC of the bivariate for DTA. Figure S2. Univariate sub-group analysis of sensitivity with random model based on gender. G represents sub-group analysis of data, when g = 0 (Female), and g = 1 (Male). Figure S3. Univariate sub-group analysis of specificity with random model based on gender. G represents sub-group analysis of data, when g = 0 (Female), and g = 1 (Male). Figure S4. Univariate sub-group analysis of DOR with random model based on gender. G represents sub-group analysis of data, when g = 0 (Female), and g = 1 (Male). Figure S5. Univariate sub-group analysis of sensitivity with random model based on gender. G represents sub-group analysis of data, when g = 0 (Asia), g = 1 (Europe), and g = 2 (Australia). Figure S6. Univariate sub-group analysis of specificity with random model based on gender. G represents sub-group analysis of data, when g = 0 (Asia), g = 1 (Europe), and g = 2 (Australia). Figure S7. Univariate sub-group analysis of DOR with random model based on gender. G represents sub-group analysis of data, when g = 0 (Asia), g = 1 (Europe), and g = 2 (Australia). [file 12938_2023_1132_MOESM1_ESM.docx]
